# Sexual and reproductive health services in universal health coverage: a review of recent evidence from low- and middle-income countries

**DOI:** 10.1080/26410397.2020.1779632

**Published:** 2020-07-20

**Authors:** T. K. Sundari Ravindran, Veloshnee Govender

**Affiliations:** a Principal Visiting Fellow, United Nations University, International Institute for Global Health, Kuala Lumpur, Malaysia; b Scientist, Department of Sexual and Reproductive Health and Research, World Health Organization, Geneva, Switzerland.

**Keywords:** universal health coverage, sexual and reproductive health, health financing, access, gender

## Abstract

If universal health coverage (UHC) cannot be achieved without the sexual and reproductive health (SRH) needs of the population being met, what then is the current situation vis-à-vis universal coverage of SRH services, and the extent to which SRH services have been prioritised in national UHC plans and processes? This was the central question that guided this critical review of more than 200 publications between 2010 and 2019. The findings are the following. The Essential Package of Healthcare Services (EPHS) across many countries excludes several critical SRH services (e.g. safe abortion services, reproductive cancers) that are already poorly available. Inadequate international and domestic public funding of SRH services contributes to a sustained burden of out-of-pocket expenditure (OOPE) and inequities in access to SRH services. Policy and legal barriers, restrictive gender norms and gender-based inequalities challenge the delivery and access to quality SRH services. The evidence is mixed as to whether an expanded role and scope of the private sector improves availability and access to services of underserved populations. As momentum gathers towards SRH and UHC, the following actions are necessary and urgent. Advocacy for greater priority for SRH in government EPHS and health budgets aligned with SRH and UHC goals is needed. Implementation of stable and sustained financing mechanisms that would reduce the proportion of SRH-financing from OOPE is a priority. Evidence, moving from descriptive towards explanatory studies which provide insights into the “hows” and “whys” of processes and pathways are essential for guiding policy and programme actions.

## Résumé

S’il n’est pas possible de réaliser une couverture santé universelle (CSU) sans satisfaire les besoins de santé sexuelle et reproductive (SSR) de la population, quelle est donc la situation actuelle vis-à-vis de la couverture universelle des services de SSR et dans quelle mesure les services de SSR ont-ils été priorisés dans les plans et processus nationaux de CSU? Telle était la question centrale qui a guidé cette analyse critique de plus de 200 publications entre 2010 et 2019. Les conclusions sont les suivantes: dans beaucoup de pays, le panier de services de santé essentiels exclut plusieurs services fondamentaux de SSR (par exemple les services d’avortement sûr, les cancers reproductives qui sont déjà peu disponibles. L’insuffisance du financement public national et international des services de SSR contribue à un fardeau persistant de dépenses à la charge des patients et à des inégalités dans l’accès aux services de SSR. Les obstacles politiques et juridiques, les normes sexuelles restrictives et les inégalités fondées sur le genre contrarient la prestation de services de SSR de qualité et l’accès à ceux-ci. Les données ne sont pas concluantes pour déterminer si un élargissement du rôle et de la portée du secteur privé améliore la disponibilité et l’accès aux services des populations sous-desservies. Alors que la dynamique s’accélère autour de la SSR et de la CSU, les mesures suivantes sont nécessaires et urgentes. Un plaidoyer est requis pour qu’une priorité accrue soit accordée à la SSR dans les paniers publics de services essentiels et que les gouvernements alignent leur budget de santé sur les objectifs de la SSR et de la CSU. La mise en œuvre de mécanismes de financement stables et soutenus, qui réduiraient la proportion de financement de la SSR à la charge des patients est prioritaire. Des éléments d’information, en passant d’études descriptives à des études explicatives, qui donnent des indications sur le fonctionnement et la raison d’être des processus et des parcours, sont essentiels pour guider les mesures politiques et programmatiques.

## Resumen

Si la cobertura universal de salud (CUS) no puede lograrse sin satisfacer las necesidades de salud sexual y reproductiva (SSR) de la población, ¿entonces cuál es la situación actual con relación a la cobertura universal de los servicios de SSR y la medida en que los servicios de SSR han sido priorizados en los planes y procesos nacionales de CUS? Ésta es la interrogante central que guió esta revisión crítica de más de 200 publicaciones entre los años 2010 y 2019. A continuación, se exponen los hallazgos. El Paquete Esencial de Servicios de Salud (PESS) en muchos países excluye varios servicios críticos de SSR (ej., servicios de aborto seguro, cánceres reproductivos) que ya están escasos. El financiamiento público inadecuado a nivel internacional y nacional de los servicios de SSR contribuye a la continua carga de gastos corrientes y desigualdades en el acceso a los servicios de SSR. Las barreras políticas y legislativas, las normas de género restrictivas y las desigualdades de género obstaculizan la prestación y accesibilidad de los servicios de SSR de calidad. La evidencia es contradictoria en cuanto a si la ampliación del rol y alcance del sector privado mejora la disponibilidad y accesibilidad de los servicios para las poblaciones desatendidas. A medida que la SSR y CUS cobran impulso, las siguientes acciones son necesarias y urgentes. Es imperativo abogar por asignar mayor prioridad a la SSR en el PESS del gobierno y en los presupuestos de salud alineados con los objetivos de SSR y CUS. Se debe priorizar la implementación de mecanismos de financiamiento estable y continuo, que reducirían la proporción de financiamiento de SSR de los gastos corrientes. Las evidencias, que varían desde descriptiva hasta estudios explicativos que facilitan comprender los “cómo” y “por qué” de los procesos y las vías, son esenciales para guiar las acciones relacionadas con políticas y programas.

## Background

At the International Conference on Population and Development (ICPD) in Cairo (1994), the global community adopted a Programme of Action, which affirmed that choice and self-determination, gender equality and human rights constitute the keystone of population policies.* Recognising that realising the right to reproductive health for all, without discrimination, is critical to exercising choice, the Programme of Action committed to achieving universal access to sexual and reproductive health (SRH) by 2015. This commitment is far from fulfilled, and the ICPD+25 summit was convened in November 2019 to mobilise the political will and the financial allocations urgently needed to do so. While substantial progress has been made in the reduction of maternal mortality and in meeting the demand for contraceptive services, and in providing antiretroviral therapy for HIV, there has been little attention to diverse health needs of vital importance such as comprehensive sexuality education, gender-based violence, safe abortion services, treatment of sexually transmitted infections and reproductive cancers. As Starrs et al (p.7)^1^ argue “Ultimately, almost all 4·3 billion people of reproductive age worldwide will have inadequate sexual and reproductive health services over the course of their lives.”

Target 3.7 of the Sustainable Development Goal (SDG) 3 on health reaffirms the ICPD commitment of universal access to SRH, extending the deadline for its achievement to 2030. However, rather than being a stand-alone commitment as in ICPD, universal access to SRH services in SDGs stands alongside Target 3.8, of achieving universal health coverage (UHC). Universal Health Coverage is achieved when *“All people obtain the health services they need – prevention, promotion, treatment, rehabilitation, and palliation – without risk of financial ruin or impoverishment, now and in the future”*.^2^

Many advocates for sexual and reproductive health and rights (SRHR) have acknowledged that progress towards universal access to SRH services would not be possible without progress towards UHC.^3^ Universal access to SRH services calls for a well-functioning health system with adequate human and financial resources, which is responsive to the needs of all sections of the population. This is also what UHC entails. Conversely, UHC can hardly be achieved if the SRH needs of the population are not met. In other words, SRH and UHC are mutually reinforcing. The global commitment to achieving both these SDG targets offers a renewed opportunity to forge new alliances to move the SRH agenda forward.

Despite the crucial role of SRH services for achieving UHC, SRH services have not always been on the agenda when global and national-level discussions and decisions related to UHC are made on financing health care, allocating resources and setting priorities.^4–7^

This paper draws on a review commissioned by the Department of Sexual and Reproductive Health and Research (World Health Organization) which aimed to explore the literature on universal coverage of SRH services within the context of UHC, to synthesise what is known and to identify the gaps. The paper focuses on lower- and middle-income countries (LMICs) for the following reasons. The first relates to relatively poorer access to and higher unmet need for SRH services in LMICs. As noted by WHO (p.2),^8^ “Sub-Saharan Africa and Southern Asia accounted for approximately 86% (254,000) of the estimated global maternal deaths in 2017”. Second, well-functioning and well-resourced health services are an important prerequisite for advancing towards UHC. However, many LMICs and especially low-income countries continue to be dependent on external funding to support their health systems. As argued by Starrs et al. (p.16)^1^ “scarce human and financial resources, and a paucity of political commitment in some cases, have prevented health-care systems in low-income and middle-income countries from offering comprehensive sexual and reproductive health services”.

The paper is structured as follows. Following this introduction, the second section presents the methodology adopted for the review. The third section presents the findings of the review in four sub-sections or major thematic areas. The thematic areas emerged through a combination of *a priori* themes informed by the research questions (i.e. deductive analysis) and an analysis of the literature (i.e. inductive analysis). The following thematic areas are presented in the findings; (1) inclusion of SRH services in essential packages of health services (EPHS); (2) financing of SRH services; (3) challenges in the implementation of SRH services; and (4) accountability mechanisms. The fourth section summarises the key findings and their implications, while the fifth section highlights gaps in the literature and presents an agenda for future research.

## Methods

The main objective of the review was to explore the current situation vis-à-vis universal coverage of SRH services, and the extent to which SRH services have been prioritised in national UHC plans and processes. This main objective translated into a series of research questions on which the literature search was based. The research questions sought to examine the range of SRH services included in the Essential Packages of Health Services (EPHS) and in the Health Benefit Packages (HBPs) of LMICs; how financing arrangements and reforms affected population and service coverage and financial risk protection for SRH services; the key implementation challenges in delivering SRH services; and accountability mechanisms and initiatives for SRH services. The reason for carrying out searches pertaining to multiple research questions was that the literature on SRH and UHC was fragmented across specific components of SRH services, and specific dimensions of UHC such as financing or EPHS.

The complex nature of the review, involving multiple research questions, answering each of which necessitated scanning a vast array of sources to identify and extract relevant information, required us to develop a suitably tailored methodology. We did not find the search processes laid out in standard guidelines such as the PRISMA suitable for our purpose. We describe in some detail below, how the search terms were identified and the search conducted.

We developed a set of search terms corresponding to each research question, combining specific components of SRH services with various aspects of EPHS, financing, implementation challenges and accountability. Appendix Table A1 presents the research questions and the search terms corresponding to each research question explored in this paper. The search was conducted combining each SRH search term with each UHC search term for all questions except the questions related to unique service delivery challenges.

The inclusion criteria for the review were :

- Publications in English;
- Published during 2010 –2019; This period was selected for two reasons. Firstly, this period includes the adoption of the SDGs and SRHR and UHC as global priorities in 2015. Second, preceding and during this period (i.e. 2010–2019), there has been significant health systems, including UHC-related, reform with implications for SRHR and correspondingly an increase in the evidence base.
- Peer-reviewed journal articles, reports, fact sheets, and working papers of multilateral and bilateral agencies, international non-governmental organisations and universities;
- Covering one or more sexual and reproductive health services;
- Covering one or more of the following dimensions of Universal Health Coverage: EPHS, HBPs, financing, implementation challenges and accountability mechanisms.

The databases searched included PubMed and Scopus, websites of multilateral organisations (WHO, UNFPA, UNOHCHR, UNICEF), The World Bank, bilateral organisations (DFID and USAID), international SRHR non-governmental organisations (NGO)s, and Google Scholar. Original articles cited in systematic reviews and useful articles from articles selected for the review were followed up and included. Although the search was not exhaustive in terms of the databases included, we are fairly certain that a vast majority of relevant articles and publications have been included, based on scanning references cited in systematic reviews and recent articles.

Two researchers scanned the abstracts, selected those that fitted the inclusion criteria and obtained the full texts. Full texts were also scrutinised by two researchers; the first researcher carried out a quick read to verify if the document fulfilled the inclusion criteria. The second researcher carried out a quality check based on the following broad criteria:

- Are the aims and objectives of the research clearly stated?
- Is the research design clearly specified and appropriate for the aims and objectives of the research?
- Do the researchers provide a clear account of the process by which their findings were produced?
- Do the researchers display enough data to support their interpretations and conclusions?
- Is the method of analysis appropriate and adequately explicated?^9^

The review included 253 publications. Unlike the case of systematic reviews, where rigorous quality standards are applied so that specific questions may be answered (e.g. “what works”? “what is the incidence of impoverishment … ”?), our purpose was to get a comprehensive overview of what is known. Therefore, we tended to be inclusive and rejected only papers that did not help answer the research questions because of poor quality.

Data was coded and analysed based on thematic analysis. The framework of analysis was based on the research questions. Codes and subsequent sub-themes were generated from the review and analysis of the literature, which were then checked against the framework. Sub-themes were named and refined and these were finally related back to the study objectives.

## Findings

### Inclusion of SRH services in Essential Packages of Health Services (EPHS) and Health Benefit Packages (HBP)

#### EPHS and HBPs

One of the biggest challenges when planning for UHC relates to how governments of LMICs with a limited budget would provide priority health care services that will be available to all its people with financial risk protection. Many LMIC governments have sought to do so by developing an “Essential Package of Healthcare Services (EPHS).”

The EPHS^†^ are defined by the countries themselves and represent the range of health services that are to be made available, usually through government health services, to all people, at no cost or minimal cost at the point of receiving the service. In practice, however, not all services mentioned in the EPHS are available in public health facilities, and users have to pay for these in private facilities. This has led to a revised, more pragmatic, definition of EPHS as a policy statement of intent and commitment, which may or may not be supported by adequate financing.

The term Health Benefit Package (HBPs) is used to refer to services that are linked to a financing mechanism such as Social or Voluntary Health Insurance to which enrolees are entitled, or available at no/subsidised charges through government or international donor funding.^10^ Inclusion of a service in an HBP is a better indicator of its availability with financial risk protection. EPHS documents tend to list broad health care needs, while HBPs have to be more precise about exactly what is covered or not covered by a specific scheme and in some instances, at what level of care.

#### Inclusion of SRH services in the EPHS of selected LMICs

**Figure 1.**
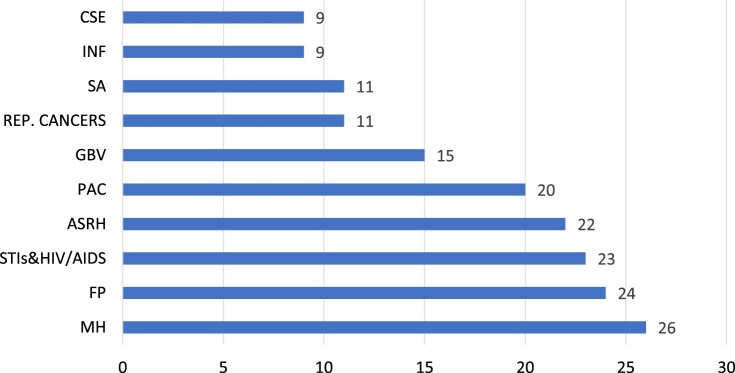
Inclusion of SRH services in EPHS packages of countries (N = 26) Source: Wright ^11–12,13,14,15,16,17,18,19,20,21,22,23,24,25,26,27,28,29,30,31,32,33,34^, Sundewall^35^ CSE - any education related to sexuality and reproduction for adolescents & young people INF - counselling, testing and management of infertility SA - safe abortion REP CANCERS - screening (and treatment) for cervical / breast cancers GBV - services related to the prevention and management of sexual and gender-based violence PAC - post-abortion care ASRH - adolescent sexual and reproductive health services STIs & HIV/AIDS - prevention and treatment, including PMTCT and ARV FP - contraceptive services MH - maternal health including emergency obstetric care

The present review found that EPHS across many countries exclude many SRH services that are already poorly available. An analysis based on 24 LMICs,^11–34^ ^‡^ shows that almost all countries included maternal health care including obstetric emergencies, and family planning services (Figure 1). Some SRH services for adolescents are included in 21 countries, mainly family planning and STI/HIV prevention and management. However, over 60% of the EPHS do not even mention services related to safe abortion, infertility, and screening for cervical and breast cancers. Comprehensive sexuality education (CSE) is effectively absent, with weak mentions of SRH awareness, education, and/or counselling. However, it is possible that CSE is not mentioned in the EPHS because it is a part of the education sector's mandate in many countries.

The mention of a specific SRH service in the EPHS may not be accompanied by all or even the essential minimum services. While the EPHS in 13 of 24 countries included gender-based violence, services for addressing intimate partner violence were specifically included in only three countries. Similarly, of the nine countries that have included reproductive cancers, only two countries include treatment for cervical and breast cancer.^11–34^

Since inclusion of an SRH service in the EPHS of a country does not guarantee the availability of the service with financial risk protection, it is important to examine whether or not an SRH service is included in the HBP of mandatory and voluntary insurance schemes, in tax-funding by the government, or donor funding. Wright and Holtz found that of 22 countries for which data were available, there was only a 30% alignment between SRH services included in the EPHS of a country and those included in the HBPs of the same countries.^10^

Two studies are the source of information on the inclusion of SRH services in countries’ HBPs. One is a review of SRH services in the UHC plans of six countries^§^ of the Asia-Pacific, and the second, a review of the inclusion of family planning in the HBPs of nine Latin American countries.^36,37^ ^¶^

Across the six Asia-Pacific countries, maternal health services, including emergency obstetric care, were covered by the insurance schemes and also available in government health facilities without user fees, at least at the primary care level. Diagnostic services and treatment for sexually transmitted infections (STIs) were fully covered in four and three of five countries, respectively. Support for HIV-related services in all countries except Thailand was from pooled external donor support. Coverage for safe abortion services was available only in Thailand, for indications that are legal in the country, and absent in some countries with relatively liberal abortion laws such as Cambodia, India and Vietnam. Contraceptive services were not included in the HBPs of two government insurance schemes, and available to a limited extent in a third.^36^

Contraceptive services were fully covered in five of the nine LAC countries in all social insurance schemes, covered for all methods except sterilisation in one, covered but largely unavailable in one and not covered in two countries. In contrast, private insurance schemes covered contraceptive services only in two countries: Chile and Colombia, and in both instances, co- payment was involved.^37^

#### Actors and processes for evolving an EPHS

An ideal EPHS should be developed through transparent and deliberative processes, in consultation with all the stakeholders, and especially the users, or civil society organisations representing users, including those from vulnerable groups.^38^ From the available evidence, the actors involved in developing EPHS included (in all but five of 16 countries for which we had the information) appear to be limited to government, international donors, and national and international technical experts. There appears to be less involvement of communities and civil society organisations (CSOs); they participated in the EPHS process of five (of 16) countries. Not much is known about how they influenced the process.^35,39^

One study provided some information on the priority-setting processes for developing the EPHS in Ghana. The study found a disconnect between SRH actors in the Ministry of Health, and the priority-setting processes for EPHS. SRH actors from the government were not invited nor sought to be involved in the EPHS process, perhaps because SRH services were mainly funded by international donors. Priority-setting was carried out based on disease-ranking by DALYs, which resulted in the inclusion of a narrow range of SRH services related to HIV, maternal health and contraception, leaving out a majority of SRH services defined in the ICPD Programme of Action.^40^

There is both a need, and scope for civil-society participation in country processes for development of EPHS and HBPs. Such participation should include the representation of the voices of the most marginalised groups (see Box 1).

Box 1: **Advocacy for the inclusion of SRH services in a National Insurance Services package.**

In Zambia, the Centre for Reproductive Health and Education (CRHE), a national NGO, was supported by Population Action International (PAI) to engage with the UHC processes in the country and to advocate for prioritising SRH services. Zambia's National Health Insurance Scheme (NHIS) has been implemented as part of progressing towards UHC, but civil society organisations (CSOs) working on SRH issues were not part of any of the consultative processes.

CRHE convened engagement which brought together government, NGOs, and medical associations to advocate for the inclusion of all existing SRH services in the NHIS benefits package. They were also able to convince the authorities to include CSO representatives in the National Health Insurance Authority’s board. As a consequence, there are now CSO representatives in various technical committees and working groups related to NHIS. They have been giving regular feedback to the authorities on the non-availability of guaranteed SRH services in specific settings and continue to be engaged in advocacy for strengthening SRH services for workers in the informal sector; and inclusion of contraceptive services and adolescent SRH education in the NHIS benefits package.

**Source**: Population Action International.^41^

The review did not find information on the criteria for inclusion of services in EPHS. A paper from Thailand, which has comprehensive coverage of SRH services in its Universal Health Coverage Scheme, proposes a decision-making framework for prioritising SRH services to be included in an EPHS, bearing in mind the budgetary implications and availability of resources. The framework examines SRH service needs, demand and supply, and proposes making a start with SRH services that are needed, demanded and available.^42^ ** This framework may be a starting point that could be suitably adapted to be used as a part of deliberative processes involving a wide range of stakeholders.

### Financing SRH services

To progress towards UHC and ensure both equitable access to health services, financial protection and overall sustainability, health services should be funded through predominantly domestic public funding which combines taxes and prepayment mechanisms.^43^ As described below, particularly in low-income countries, domestic public funding of SRH services remains a challenge, contributing to inequities in financing and access to SRH services.

#### Overall patterns of SRH financing

Most countries rely on a mix of different sources of revenue to finance the health system. Sources of revenue include government taxes; external funding by bilateral and multilateral donors and private foundations; mandatory social insurance schemes, which raise revenue from payroll deductions of employees and contributions from employers; voluntary insurance schemes; and payment by households at the point of seeking care, known as out-of-pocket expenditure (OOPE).

**Figure 2.**
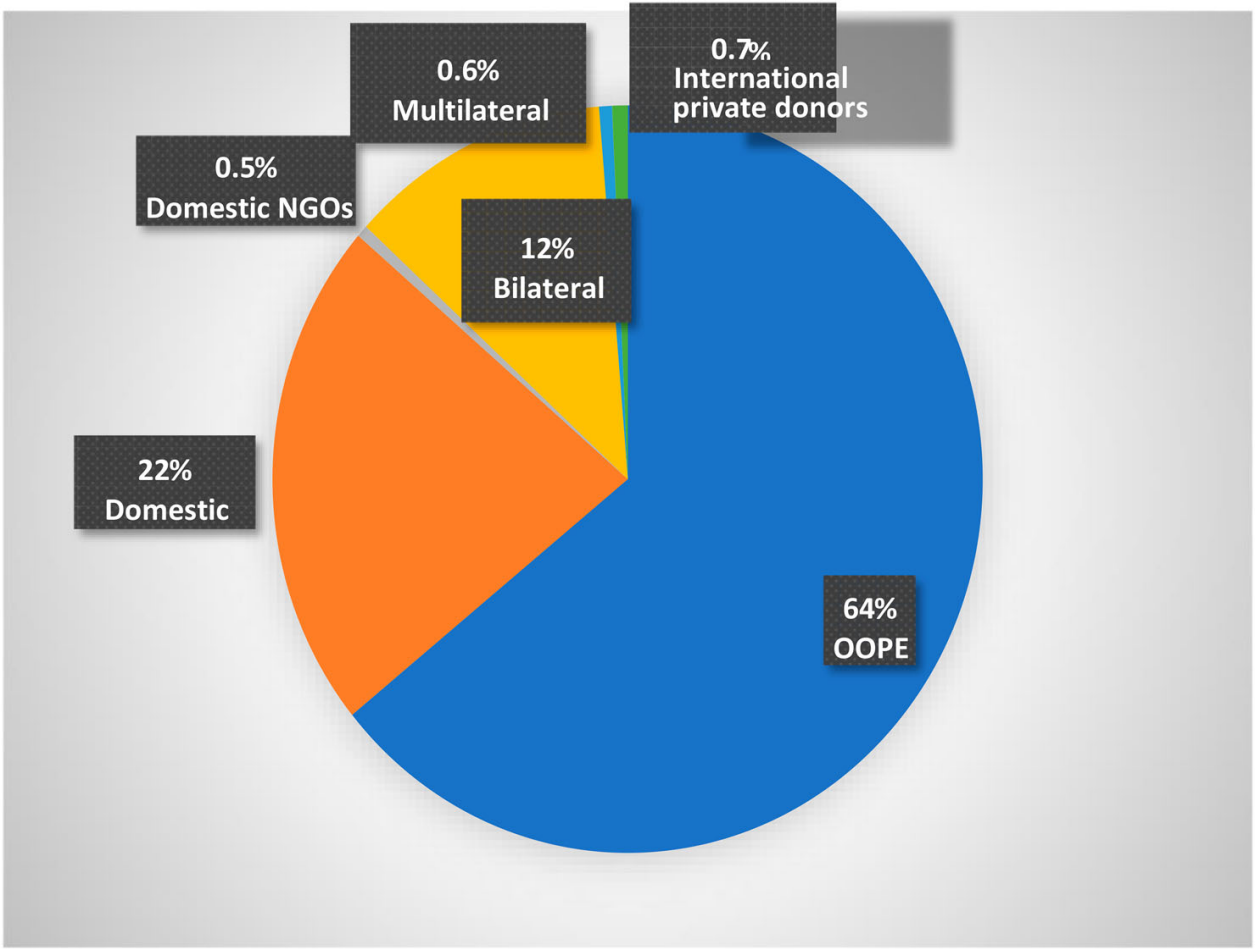
Financing of the ICPD costed package, 2012. Source: Drawn based on data from IPPF.^20^

Information on aggregate patterns of SRH financing is available for a limited range of SRH services, known as the “costed package”^††^ of ICPD services. In 2012, almost two-thirds of all expenditure on SRH services in developing countries was from out-of-pocket expenditure by households.^44,45^ Government spending accounted for about 22%. International public funding from bilateral and multilateral sources was around 13.5%. This aggregate picture holds in general, except in some countries which are heavily dependent on donors (Figure 2).

In terms of national patterns of funding, information from nine countries on financing reproductive^‡‡^ health services showed considerable inter-country variations.^47–50^ OOPE was the largest source of RH financing (as seen in the overall pattern for developing countries) in three countries (Kenya, 38%; Uganda, 48% (2006); Burundi, 43% (2007)). International public funding contributed most to SRH financing in three countries in 2006 (Ethiopia, about 45%; Malawi, 57%; Rwanda; 80%). Government funding was the main source of RH financing in Mexico (44.3% in 2012), the Indian states of Bihar (59%) and Karnataka (76%) in 2010–2011 and in Tanzania (45% in 2006). Social and private insurance schemes contributed to less than 5% of RH financing in 2006 in six sub-Saharan African countries,^47^ while in Mexico, social security funds from social insurance schemes for formal sector employees accounted for 37% of RH financing in 2012.^50^ The unit cost of providing modern contraception and meeting unmet need for family planning in developing countries was estimated to be US$16 in 2003, and yet most study countries spent less than US$16 for *all* RH services and commodities, with Ethiopia’s spending as low as US$4.^47^

#### Out of pocket expenditure

From the perspective of UHC, OOPE is the least desirable mode of financing. It makes access to healthcare contingent on ability to pay at the time of illness and acts contrary to the UHC objective of “financial risk protection”. OOPE is inequitable since households and individuals without significant disposable income and savings might not be able to afford health services even when there is need.^51^ For reasons of gender and claims over household resources and spending decisions, OOPE typically limits women's access to health care. According to WHO (p.23),^43^ “[w]omen incur more out-of-pocket payments than men … paying for delivery care and other reproductive health services places a higher financial burden on women … [and] out-of-pocket expenditure may prevent more women than men from utilizing essential services”. To move towards UHC, countries have to reduce the proportion of OOPE in total health spending, moving towards pre-payment mechanisms such as tax-funding and Social Health Insurance.

A large number of studies show that high OOPE for obstetric emergencies and complications of unsafe abortion, when safe abortion services are not available, results in catastrophic health expenditures.^52–59^ Even where there is fee waiver, non-medical expenditure, informal payments and expenditure on drugs, consumables and diagnostics result in high OOPE.^60–73^ The incidence of catastrophic health expenditure is found to be much higher among the poorest sections of the population,^60,61,64,65,69^ contributing to widening inequities in health. However, there is very limited evidence on the OOPE and catastrophic health expenditure relating to SRH. One of the studies that have tracked this expenditure over time is from India, focusing on free maternal health care (see Box 2).^65^

Box 2: **Tracking out-of-pocket expenditure and catastrophic health expenditure relating to free maternal health care in India**

The study compares OOPE and catastrophic health expenditure (CHE) for 2004 and 2014 using data from the National Sample Surveys. In 2011, the government of India introduced the *Janani Shishu Suraksha Karyakram* (JSSK), which provides free pregnancy, delivery, and post-partum and neonatal care, including emergency obstetric care and caesarean-sections and emergency neonatal care. The programme also covers transportation and food costs. This was in addition to a conditional cash transfer programme for institutional deliveries, the *Janani Suraksha Yojana*. The study found significant increases in the utilisation of maternal health care and steep declines in the incidence of CHE. Utilisation of antenatal care, institutional delivery and post-natal care increased from 75.4% to 90.4%; 43.3% to 82.6% and 64.4% to 79.3% respectively. The incidence of catastrophic health expenditure declined from 56.2% to 29.4% for households where the women had exclusively used public health facilities for all ANC, institutional delivery, and PNC. The incidence of CHE declined for all income quintiles except the poorest; however, there was a significant gap in the incidence between the poorest and richest households. While 74.6% of the poorest households using maternal health services from public health facilities experienced catastrophic health expenditure in 2014, only 0.4% of the richest households did. The comparable figures for households using private maternal health care were 95.8% and 20.7%, respectively.

**Source**: Mohanty and Kastor.^65^

#### International funding

International public funding has been an important source of SRH financing especially in low- and lower-middle income countries. USAID accounts for the major share of SRH funding, and in 2012, contributed to 57% of the total development assistance towards the ICPD costed package.^74^ Dependence on USAID funds has resulted in much volatility in funding received for SRH services. This is because during every Republican Presidency since the mid-1980s, funding from USAID is subject to the condition that the recipient will not carry out any activities related to the provision of safe abortion services.^75^

Further, while the overall funding for the ICPD “costed package” increased, most of the funding was for HIV/AIDS. Between 1994 and 2014, international public funding for the ICPD costed package increased from US$0.6 to US$7.0 per woman of reproductive age living in developing countries. However, 65% of the funding was for HIV/AIDS and STIs (2012). The share of family planning services was only 9% and had fallen steadily as a proportion of international funding between 1994 and 2012.^74^ Moreover, international SRH funding to individual countries could fluctuate wildly. For example, Kenya received US$15.9 million as international contribution for SRH services in 2005, which fell to US$0.5 million in 2007 and rose again to US$14.5 million in 2010.^76^

To address the funding gap, global initiatives such as the Global Financing Facility (GFF) support national ministries of health to identify and scale-up high impact, cost-effective interventions that can be sustainably funded through a combination of increased domestic and international funding.^77,78^ Although blended financing, and specifically GFF, has enabled the inclusion and prioritisation of SRH services, several concerns have been raised. These include the focus on maternal health and family planning services and the exclusion of other critical (e.g. safe abortion, sexual and gender-based violence) services.^79^ In addition, in the absence of viable domestic public funding, the focus on domestic financing can shift the burden of financing SRH services and products to OOPE by the poorest and most vulnerable groups.^45,80^

#### Government funding

Government funding is the second-most important source of SRH financing in many LMICs. Because of the limited number of countries with RH sub-accounts, information on government-funding for SRH services is scarce. Available evidence from nine countries suggests that the proportion of government funding for SRH can fluctuate from year to year, even when the overall health budget increases. For example, government contribution to SRH financing increased in three countries: in Burundi, from 15% in 2010 to 19% in 2012;^48^ in Kenya, from 34% in 2005–2006, to 40% in 2009–2010^76^ and in Mexico, from 11.4% in 2003 to 44.3% in 2012.^50^ It decreased in Rwanda, Tanzania, and Malawi during 2002–2006.^47^

A study from Mexico shows that increased spending and allocation favouring households without social security resulted in greater financial risk protection, especially to poorer households.^50^ During 2003–2012, there was a three-fold increase in government expenditure on health and a steep decrease in OOPE as a proportion of total expenditure (from 51.5% in 2003 to 16.3% in 2012). This was the period immediately following the introduction of health reforms to enrol pregnant women without social security coverage in a social health protection scheme (*Seguro Popular* or SP), which entitled them to free services at the point of care. OOP spending on childbirth and complications reduced by over 80% for households without social security. Comparable figures for antenatal care and family planning were 61% and 64%, respectively. OOPE for all these services also decreased for households with social security, but the reductions were modest when compared to households without social security.

#### Health insurance

Health insurance is a pre-payment mechanism that allows for pooling revenue across people at high and low risk of having a healthcare need. Mandatory insurance schemes such as the deduction of a fixed proportion of the salary also allow for cross-subsidising across income groups. The intention is to enable access to health care irrespective of the ability to pay at the time when a healthcare need arises, with financial risk protection. There are very few studies directly addressing the issue of SRH services in public, private, and community-based insurance schemes, and this represents a major evidence gap.

Studies from HIC show that many SRH services (e.g. contraception and abortion services) may be excluded from private insurance plans.^81^ Another study observed that the exclusion of safe abortion services from Medicaid following the Hyde Amendment in 1977 has resulted in delays in seeking abortion services, catastrophic health expenditures, and impoverishment, or unintended pregnancies carried to term.^82^ However, there are few studies on the coverage of SRH services by public and private insurance schemes in LMICs.

The design of insurance schemes and the process of enrolling beneficiaries may give rise to inequities in access to insurance coverage. Moreover, the exclusion of many SRH services including those that women need to protect their health and wellbeing is discriminatory. A study of Rwanda’s Mutual Health Insurance scheme found that female-headed households across all income quintiles were two-thirds less likely to have Mutual Health Insurance.^83^ Experience with voluntary private insurance in South Africa revealed intersectional inequalities based on gender and other axes of vulnerability. The heads of partially insured households^§§^ were more likely to be female, unmarried, with primary-school education or no education, unemployed, and Black.^84^ Encouragingly, however, studies from Ghana on its National Health Insurance Scheme, which covers all sections of the population, report a higher female than male enrolment.^85,86^ The higher enrolment may be the result of a waiver of premiums for pregnant women and the indigent, a majority of whom were women.^87^ Providing premium exemptions may be the route to ensuring the enrolment in insurance schemes of groups without access to cash incomes.^88^ However, the extent to which premium-exemptions would result in increased utilisation may depend on whether affordability is a major barrier to accessing the specific SRH service under consideration.^89^

#### Demand-side financing mechanisms

Many LMICs have introduced demand-side financing (DSF) mechanisms to remove financial barriers to accessing health care services and to reduce OOPE. The objective is to increase the use of essential health services by the poor and under-serviced groups. The services usually covered by DSF are those with significant externalities such as family planning, maternal healthcare, and immunisations.^90^ Some examples of DSF mechanisms include conditional cash transfers (CCTs), voucher schemes and publicly funded health insurance schemes (PFHIS) for the poor and indigent. An implicit assumption underlying DSF mechanisms is that financial barriers, including transportation and food costs, are the most important reason for underutilisation of services.^90^ While many of the DSF mechanisms have improved overall access to services, the benefits of these mechanisms often elude the poorest and the most vulnerable populations.

Evidence suggests that voucher schemes improved access to maternal health services and FP and also reduced OOPE.^91–94^ Conditional cash transfers have been found to increase utilisation of maternal and neonatal health services,^95–97^ but not family planning services.^98^ Studies have found that CCTs may fail to benefit the most marginalised or provide financial risk protection for all.^99–101^ and their impact on improving health outcomes has been mixed.^95,96,98^ PFHIS for the poor seem to experience gender-based barriers to enrolment, similar to other insurance schemes.^102–104^ Further, design elements such as limiting enrolment to five members of a household, and a fixed financial coverage which is allocated by the households to its members, may result in the exclusion of the less powerful household members such as women and the elderly from enrolment or use of insurance benefits.^105^

### Implementation of SRH services

The review examined the literature on service-delivery challenges unique to (or predominantly affecting) SRH services (i.e. policy and legal barriers and restrictive gender norms and gender-based inequalities) and the role of the private sector as an implementer.

#### Legal and policy barriers

Legal and policy barriers that deter access to SRH services affect a significant proportion of the world’s population. The requirement of parental or spousal consent poses a barrier to contraceptive services in about 5–10% of the world's countries,^106^ and two-thirds of the women live in countries where there are one or more restrictions as to the reasons for seeking safe abortions.^107^ The other side of the coin to forfeited use is coerced or involuntary use, prevalent in the case of sterilisations for vulnerable population groups such as persons living with disabilities,^108^ which erode users’ trust in the health system and can affect SRH service utilisation as a whole. The criminalisation of same-sex sexual conduct and sex-work^109,110^ and barriers to legal gender recognition for transgender people enhance the exposure to health risks of these groups.^111^ They also result in leaving vulnerable populations out of the realm of accessible and affordable SRH care.

#### Restrictive gender norms

Restrictive gender norms influence behaviours related to sexuality and reproduction, and alongside gender-based inequalities in access to resources and decision-making, they often erect barriers to the use of health services by women,^112–114^ men^115,116^ and transgender persons.^117–120^

An important dimension of gender norms and inequalities is gender-biased attitudes and practices in the health system, which remain important barriers to effective and timely use of SRH services. Some of these practices include requiring spousal consent for providing contraceptive and abortion services^121^ and denial of services to adolescents and to young unmarried persons (even in the absence of legal restrictions). Among adolescents and young unmarried women and men, lack of privacy and confidentiality; provider attitudes disapproving of sexual activity; and provider-disclosure to parents were major deterrents to access to SRH services.^122,123^ LGBTQ persons frequently experienced discriminatory hostile behaviour and “micro-aggression” from health providers and were at times treated as mentally unstable because of their “deviant” behaviour.^124–126^ Disrespect and abuse of women seeking delivery or abortion services is an extreme manifestation of gender-biased attitudes and impacts negatively on the use of institutional delivery and safe abortion services.^127–130^

#### Challenges of private sector engagement

Practically all LMICs have mixed health systems, with the public-private mix varying in nature and extent, both in financing and in service delivery. One of the critical challenges for countries is to identify and ensure an appropriate role for the private sector that aligns with and does not detract from the country's UHC goals.

In the case of SRH service delivery, the private sector accounts for a large share in many LMICs.^131,132^ Since the late 1990s, many international donors and the International Finance Corporation (IFC) of the World Bank, and more recently, the GFF, have made deliberate and concerted efforts to engage the private health sector in SRH service provision.^77,133^

The most common modes of private sector engagement found in the literature are (1) contracting with private sector SRH service providers, (2) social franchising networks, (3) social marketing programmes and (4) voucher schemes. All four are aimed at expanding availability and access to essential health care for underserved populations by expanding the pool of providers and service delivery points. Voucher schemes are primarily intended to provide financial risk protection (discussed earlier in the paper). To what extent do these modalities of engagement with the private sector expand coverage and utilisation especially for the underserved populations, offer good quality services with financial risk protection?

Systematic reviews of the outcomes of various modes of private sector engagement for the provision of SRH services highlight that the quality of the evidence is weak and that only tentative conclusions may be drawn based on the evidence. We may surmise from the descriptions of these four modalities that, with the exception of social marketing, none of these modalities may increase the population or service coverage, because they often provide a narrow range of SRH services such as maternal health and family planning, through existing service delivery points. Of the four, social marketing schemes are found to increase access to knowledge, services and products,^134,135^ while voucher schemes increase utilisation as well as decrease OOPE.^91–94^ The evidence on increased utilisation through social franchising and contracting with private providers is mixed.^136–147^ Studies also suggest that the design of social marketing, social franchising, and contracting with the private sector may not be appropriate to reach the most marginalised groups.^136,146–150^ However, voucher schemes that were designed to serve specific under-served populations (e.g. adolescents, sex workers, persons living in post-conflict situations) succeeded in doing so.^151,152^ We do not know enough about the quality of care provided through the different modalities of private-sector engagement. An issue of major concern is the increasing investments by governments and especially international donors in these modalities of engaging the private sector in the absence of robust evidence about their contribution to UHC goals.^153^

The most immediate priority appears to be more robust research which takes a whole-of-health-system view and examines the nature of and interaction between the public and private sectors within the health system as a whole, which influence private sector performance and the sector's alignment with UHC goals.^154^

### Accountability mechanisms towards universal coverage of SRH services

For more than two decades, the SRHR community globally has witnessed slow progress and uncertain gains in achieving the ICPD goals. The SDGs offer a new window of opportunity to move forward, and effective accountability mechanisms to monitor progress are an important means to achieving universal coverage of SRH services. Studies show that there are several accountability mechanisms for SRH services at the international, national, and sub-national levels.

At the international level, human rights treaty bodies and the Human Rights Council^¶¶^ have played an important role in holding governments accountable for improving SRH services including maternal health, abortion and family planning, through recommendations to States on periodic reports by State Parties, and on individual complaints received by the treaty bodies.^155^ Recommendations on SRHR by the Human Rights Council (HRC) based on Universal Periodic Reviews of adherence by State Parties to human rights standards have been implemented by many countries, making the Universal Periodic Review a useful accountability tool.^38^

Many voluntary networks of international entities (e.g. Countdown 2015 and FP 2020) have been tracking resource flows for Reproductive, Maternal, Newborn, Child and Adolescent Health and Nutrition (RMNCHA-N), or specific areas such as maternal health, family planning, and STIs/HIV.^156^ More recently, government commitments to improving RMNCHA-N are being monitored on the basis of a framework to monitor-review-act proposed by the Commission on Information and Accountability (CoIA) for Women’s and Children’s Health.

At the national and sub-national level, there are examples of initiatives for examining performance accountability,^157,158^ social accountability^156,159,159–160^ as well as legal accountability.^161–162^ *** Most of the studies reviewed were descriptive, and only some provided information on the outcomes. In general, the studies did not analyse reasons for success or lack of it.

Overall, performance accountability initiatives enabled health system action at the district level to improve performance,^157,158^ and social accountability initiatives showed an increase in service utilisation and greater user-satisfaction with services.^163–164,165^ Legal accountability mechanisms in India used for seeking individual redress, were able to bring about policy changes setting quality standards for government’s maternal health and family planning services.^161,162^ Some social accountability initiatives have also improved the capabilities of women from marginalised populations to demand accountability, an outcome with potentially more long-term impact than immediate gains in quality and availability of services.^159,160^

An important learning is that accountability mechanisms and initiatives are context-specific and dependent on the democratic space available, the relationship between the state and civil society actors, the extent to which health systems are responsive to the concerns of service-users and the extent to which gender and SRHR are prioritised both in state policies and in civil society activism.^166^ Therefore, “best practices” cannot be transferred from one setting to another without suitable adaptation.

Despite the vast potential of SRHR accountability mechanisms, the picture that emerges is one of uncoordinated actions by multiple actors with a diversity of interests and capabilities. There is no information on any efforts at coordination and building synergies across international and national accountability initiatives, across civil society and government initiatives, and even among social accountability initiatives by civil society actors.^162^

From the perspective of accountability for universal coverage of SRH services and SRHR at the country level, a starting point would be to know more about the contexts in which accountability mechanisms and initiatives operate and factors influencing their effectiveness and to explore ways in which synergies may be built for effective enforcement of accountability for universal coverage of SRH services.

## Conclusions and the way forward

The review raises many concerns related to the current pattern of financing SRH services, which are detrimental to UHC goals. These include the low per capita spending on SRH services and the high levels of OOPE and catastrophic health expenditure in the financing of SRH services. Implementing financing mechanisms that would reduce the proportion of SRH-financing from OOPE is an urgent priority. Increased international and domestic public funding for a comprehensive range of SRH services is needed. Such funding has to be stable and sustained over a period of time. Advocacy for greater priority for SRH services in government health budgets is needed, including but not limited to maternal health and family planning.

There is both a need, and scope for civil-society participation in country processes for development of EPHS and HBPs. Such participation should include the representation of the voices of the most marginalised groups.

From the perspective of advancing universal coverage of SRH services, we do not know enough about the potential of engagement with the private health sector. We need to understand the effect of private sector engagement in SRH service provision on the health system as a whole, and its overall contribution to population and service coverage, and financial risk protection. From what we know thus far, dependence on the private for-profit sector for the delivery of SRH services would pose financial barriers to those from marginalised sections. Also, services that are not profitable to provide, such as safe abortion services, may not be available from the private for-profit sector, jeopardising the comprehensiveness of SRH services. It may be prudent to wait until robust evidence is available, before making further investments on specific modalities of engagement with private sector providers.

Beyond technical tasks such as mobilising resources and setting priorities, addressing policy and legal barriers to SRH, and gender biases and issues of stigma and discrimination within SRH service provision should also be on the agenda of action towards universal coverage of SRH services. Effective enforcement of accountability for SRHR calls for better coordination and innovative strategising among like-minded actors across all levels. Women’s movements and CSOs in many LMICs have engaged in such work and are allies in this project.

## Gaps in evidence and directions for future research

This review indicates significant gaps in the evidence on SRH services in UHC, despite a relatively large number of publications. There is a clustering of studies around specific themes (i.e. maternal health and family planning), while many areas (i.e. access to safe abortion services, adolescent SRH, STI, and breast and cervical cancer) remain poorly explored and understood. Of all dimensions of UHC, there is relatively more evidence including systematic reviews on financing SRH services and specifically financing arrangements for maternal health and family planning and on OOPE and (to a smaller extent) catastrophic health expenditure, while little is known about other financing mechanisms and their impact on access to SRH services.

Given the increasing prominence of the private sector in SRH services, robust studies which assess the scope of various modalities of engagement with the private sector and their impact on population and service coverage and financial risk protection for the population as a whole are urgently required.

There is a need for more rigorous studies of the outcomes of specific financing (e.g. Global Financing Facility) and service provision initiatives, and effective accountability initiatives. Many studies are based on a cross-sectional design that precludes attribution of causality or is before-and-after studies without a comparison group.

There is an urgent need to go beyond descriptive and exploratory studies. Explanatory research - which provide insights into the “hows” and “whys” of the contextual factors (i.e. political, economic and social context) and the processes and pathways that helped, or have the potential to help, a country advance towards UHC and universal access to SRH services – are required to aid policy and programme planning and implementation.

To conclude, only a small proportion of the vast technical literature on UHC has any reference to SRH, or to engaging with and examining the concepts of gender, rights and discrimination. There is a separate body of literature from the SRH world, on the importance of prioritising SRH in national UHC plans, but only a small proportion of these throw light on the extent to which LMICs have realised the prioritisation of SRH services in UHC or describe country experiences of doing so. This requires engagement within the SRH literature with concepts central to UHC such as progressive realisation^167^ and progressive universalism,^168^ and moving away from fragmenting SRH into silos (e.g. family planning, gender-based violence, maternal health care, etc.) towards comprehensive SRH which considers the unique SRH service needs of girls and women across the over the life course.^169^ There is a lengthy and unaddressed research agenda that demands urgent attention.

## Footnotes

* Principle 1 and Principle 4, respectively, of the ICPD Programme of Action, uphold human rights and gender equality and equity and women’s empowerment. UNFPA (1995). *International Conference on Population and Development – ICPD. Programme of Action*. A/CONF.171/13/Rev.1. http://www.unfpa.org/public/home/publications/pid/1973

† EPHS also referred to as core, on minimum or basic packages, may also be represented by standards of care to be provided at different levels of the health system. A country may have more than one EPHS, corresponding to different vertical health programmes.

‡ For this paper, we examined 24 case studies of the inclusion of SRH services in EPHS, authored by Wright 2015. Each of the case study documents provided the policy document on EPHS as Annex A. We mapped the SRH services mentioned for each country against the ICPD Programme of Action package of SRH services (UN 2004). To this, we added any additional countries and information in Sundewall.^35^ There was an overlap of four countries between the two studies, and Eswatini and S. Africa were the additional countries added on. The countries referred to in figure 11 are for 26 countries: Afghanistan, Bangladesh, DR Congo, Eswatini, Ethiopia, Ghana, Haiti, India, Indonesia, Kenya, Liberia, Madagascar, Malawi, Mali, Mozambique, Nepal, Nigeria, Rwanda, Senegal, South Africa, South Sudan, Tanzania, Uganda, Yemen and Zambia. We have counted even a mention of a particular area of service as "included" in the EPHS.

§ Countries included Bangladesh, Cambodia, Indonesia, Mongolia, Thailand and Vietnam.

¶ Countries included Chile, Colombia, Costa Rica, Dominican Republic, Guatemala, Haiti, Honduras, Jamaica, and Peru.

** “Need” is defined on the basis of DALYs lost for major SRH conditions, and arranged in order of DALYs lost, from the highest to the lowest. “Demand” was what people asked for and assessed on the basis of SRH service utilisation data from private health facilities. Supply was defined as the range of services included in Thailand’s HBP for the Universal Health Care (UHC) programme.

†† The ICPD “costed package” included only a limited number of SRH services based on data availability. The package did not include all interventions or all costs necessary to achieve the ICPD goal of universal access to SRH services. For example, adolescent sexual and reproductive health services; screening and management of reproductive cancers; infertility treatment, and even obstetric emergencies are not a part of the costed package.^46^

‡‡ Data pertain to reproductive health services and not sexual and reproductive health services.

§§ Partially insured households were those with only some members of the household covered under the insurance scheme.

¶¶ Some human rights treaty bodies related to SRHR include the Commission on the Elimination of All Forms of Discrimination Against Women (CEDAW), Child Rights Committee (CRC) and the Committee Against Torture and Ill-treatment (CAT). The Human Rights Council (HRC) is concerned with all human rights concerns, including those related to SRHR.

*** Performance accountability refers to a government holding the health sector answerable for delivering quality care, through measures such as quality assurance, death reviews, surveillance systems, staff performance review, and disciplinary measures. In social accountability, citizens/communities hold the service providers and the government to account. Community monitoring and scorecards, public-hearings, citizen monitoring of health facilities, and budget-tracking are some examples. Legal accountability is about holding governments accountable for violations of human rights or for not upholding the constitutional rights of citizens. Strategic litigation is one of the commonly used interventions to enforce legal accountability.

## Appendix

**Table A1.** Research questions and search-terms

Research questions	Search terms related to SRH components	Search terms related to UHC dimensions
1. What are the key themes addressed by the SRH literature on UHC?	Sexual and reproductive health services Sexual and reproductive health Sexual and reproductive health and rights	Universal Health Coverage
2. Which SRH services have been included in EPHS of different LMICs? What were the processes through which they were included?	Maternal health services; emergency obstetric care Safe abortion services; post-abortion care Family planning; contraceptive services Adolescent sexual and reproductive health services; comprehensive sexuality education Gynaecological services; infertility treatment Services for gender-based violence Cervical cancer screening; breast cancer screening; cervical cancer treatment; breast cancer treatment	Essential package of health services; essential services packages; basic services packages; Health benefit packages Priority-setting
3. What are the major sources of financing for SRH services? How does this pattern of financing affect population and service coverage and financial risk protection?	Same as above	Financing mechanisms National Health Accounts; SRH sub-accounts; User fees; Social Health Insurance; national health insurance; community-based health insurance; micro-insurance; Conditional cash transfers; Out of pocket expenditure; Catastrophic health expenditure
Research questions	Search terms related to SRH components	Search terms related to UHC dimensions
5. What has been researched on some service delivery challenges unique to/ predominantly affecting SRH services such as restrictive gender norms and gender inequality? Restrictive legal and policy environment? Private sector as a major service provider?	Gender norms; gender inequality; abortion laws; LGBTQI and legal status / criminalisation; disrespect/abuse and maternity; obstetric violence; coercive/ forced sterilisation; third-party authorisation; adolescents; stigma; discrimination; Contracting with private providers; Social marketing; Social franchising; Voucher schemes (for each of the SRH components presented above)	
4. What are the accountability mechanisms that have been used w.r.t. to universal access to SRH services at the international, national, and sub-national levels? What were the issues addressed? Who were the actors involved, and what were the processes? What were the outcomes?	Same as above	Accountability Performance accountabilitySocial accountabilityLegal accountability
